# First Molecular Characterization of Sheep Pox Viruses in Northern Ghana, 2023

**DOI:** 10.3390/v17070875

**Published:** 2025-06-21

**Authors:** Theophilus Odoom, Richard Kwamena Abbiw, David Livingstone Mawuko Blavo, Sherry Ama Mawuko Johnson, Patrick Ababio, Spencer Dugbartey, Irene K. Meki, Tirumala B. K. Settypalli, William G. Dundon, Charles E. Lamien

**Affiliations:** 1 Accra Veterinary Laboratory, Veterinary Service Directorate, Accra LG-25, Ghana; ginolapaatee@gmail.com; 2 School of Veterinary Medicine, University of Ghana, Legon, Accra P.O. Box LG139, Ghana; 3 West African Centre for Cell Biology of Infectious Pathogens, University of Ghana, MR36+W5R, Accra LG-25, Ghana; abbiw.richardk@gmail.com; 4 Veterinary Service Department, Ministry of Food and Agriculture, Navrongo P.O. Box 25, Ghana; davidlivingstoneblavo@gmail.com (D.L.M.B.); spencerkish41@gmail.com (S.D.); 5 Animal Production and Health Laboratory, Joint FAO/IAEA Centre of Nuclear Techniques in Food and Agriculture, Department of Nuclear Sciences and Applications, International Atomic Energy Agency, Wagramer Strasse 5, P.O. Box 100, A1400 Vienna, Austria; i.meki@iaea.org (I.K.M.); t.b.k.settypalli@iaea.org (T.B.K.S.); w.dundon@iaea.org (W.G.D.); c.lamien@iaea.org (C.E.L.)

**Keywords:** sheep pox virus, samples, Ghana, phylogenetic analysis, outbreak

## Abstract

Sheep pox (SP) is a contagious viral disease affecting sheep, characterized by fever, respiratory distress, hypogalactia, and skin lesions. In response to a series of outbreaks of pox-like lesions with morbidity (75%) and mortality (37%) rates among sheep in the Upper East Region of Ghana, nasal samples were obtained from affected sheep for diagnosis and characterization. The DNA extracted from these samples was tested using quantitative PCR (qPCR). Positive samples were subjected to further analysis for poxvirus marker genes using conventional PCR. Positive amplicons were sequenced, and phylogenetic analysis was conducted. The characterization and comparison of RPO30, GPCR, EEV glycoprotein, and B22R genes with other isolates demonstrated a close genetic relationship with sheep poxviruses (SPVs) identified in other African and Asian countries. This study represents the first comprehensive characterization of SPV in Ghana, and the data generated will be of significant interest to national and regional veterinary authorities.

## 1. Introduction

The sheep poxvirus (SPV), previously abbreviated as SPPV, is an enveloped double-stranded DNA virus with a genome size of 151 kb. It is classified under the species *Capripoxvirus sheeppox*, genus *Capripoxvirus*, subfamily *Chordopoxvirinae*, and family *Poxviridae* [1,2]. Other members of this genus include the goat pox virus (GPV), previously abbreviated as GTPV, and the lumpy skin disease virus (LSDV) [1,2,3,4]. These viruses are designated as a notifiable and category A contagious disease by the World Organization for Animal Health (WOAH) [5,6] and have significant implications for international trade owing to their potential to cause substantial economic losses for farmers in endemic regions [1,7]. SPV is antigenically related to GPV and LSDV, rendering them indistinguishable [8]. SPV and GPV isolates from most regions are considered host-specific [8]. However, there is growing evidence of the potential for cross-infection between sheep and goats [1,5,8,9,10,11].

SPV is primarily transmitted through direct contact with pox lesions and secretions from infected animals [5,11]. Additionally, fomites and mechanical transmission by insects, such as stable flies *Stomoxys calcitrans*, have been suggested as potential vectors [4,12]. Clinically, sheep pox is characterized by symptoms such as fever; difficulty in breathing; nasal inflammation; conjunctivitis; excessive salivation; pox lesions, particularly on un-wooled skin; and decreased milk production [1,13,14,15,16]. Lesions may appear in the vulva, lungs, and digestive tract, potentially leading to complications such as abortion and diarrhea [4,15,16]. Clinical diagnosis of sheep pox typically relies on the presence of pathognomonic skin lesions, which resemble contagious pustular dermatitis and are similar to urticaria in severe cases [4,17]. The clinicopathological presentations may mimic conditions such as variola, ectromelia, and myxomatosis [18].

The definitive diagnosis of SPV is achieved through molecular techniques, including real-time PCR, which is considered the gold standard, as well as restriction endonuclease analysis and PCR–Restriction Fragment Length Polymorphism (PCR-RFLP) [3,9,13,19]. Diagnostic samples include full skin thickness biopsies, vesicular fluid, scabs, skin scrapings, lymph node aspirates, whole blood, and nasal swabs [4,20]. Currently, there is no specific treatment for SP [7,16]; however, certain management strategies have demonstrated positive outcomes, such as symptomatic treatment, prophylaxis to prevent secondary infections, and the provision of a balanced diet [4]. Vaccination is considered a major preventive measure [7,13,16,21,22,23,24,25]. Sheep pox disease (SP) is endemic in regions such as the Middle East, Central and Northern Africa, Central Asia, India, and parts of China, with sporadic outbreaks documented in South-Eastern Europe [13,25,26]. Although there have been sporadic unconfirmed reports of SPV and GPV in 2016 [6], 2018, and 2019 [27], the etiology and epidemiology of SP, as well as the molecular characterization of SPV, have not been investigated in Ghana. Therefore, this study presents the first report of SPV associated with an outbreak of SP in the Kassena-Nankana District of the Upper East Region of Ghana in 2023.

## 2. Materials and Methods

### 2.1. Outbreak Investigation

**Figure 1:**
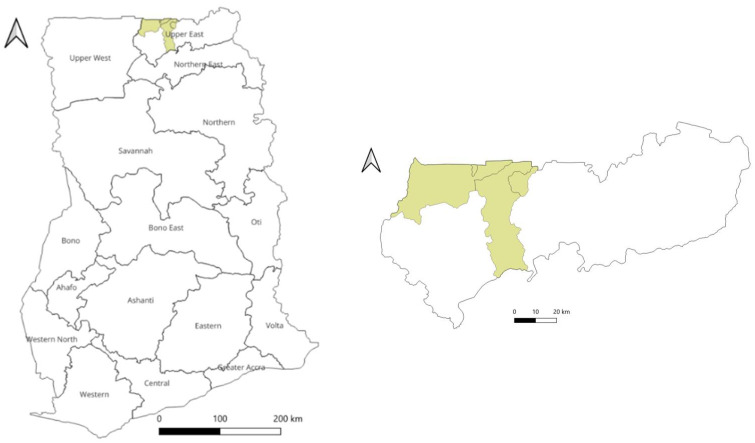
Map of Ghana (on the **left**) showing the Kassena-Nankana Municipality in the Upper East Region (on the **right**).

Between July and August 2023, the Municipal Veterinary Officer in Kassena-Nankana Municipality, located in the Upper East Region of Ghana (Figure 1), reported an increasing incidence of sheep exhibiting clinical signs consistent with SP, ectoparasite infestation, and cutaneous erythema. These reports predominantly originated from the communities of Bonia, Nagalakenia, Dawba, Kwowagnia, Vuvania, and Gigabnia, with few reports from other areas within the municipality. Investigations were conducted on the affected farms, and the sheep underwent physical examination. All farms were multispecies and housed cattle, sheep, and goats. Furthermore, all sheep within these herds were vaccinated against Peste des Petits Ruminants.

### 2.2. Sample Collection and Preparation

Twelve (12) nasal swab samples were obtained in tubes without media from 10 clinically sick and 2 apparently healthy sheep selected from sheep farms in Vunania. The swabs were kept on ice (4–8 °C) and transported to the Accra Veterinary Laboratory (AVL) of the Veterinary Services Directorate (VSD) for analysis within two days. On arrival at AVL, the swabs were resuspended in 1 mL of 10% phosphate-buffered saline (PBS) and vortexed, and the supernatant was transferred into sterile 2 mL cryotubes. The samples were stored at −20 °C until laboratory testing.

### 2.3. DNA Extraction and qPCR

Viral DNA was extracted using the QIAwave DNA Blood & Tissue Kit (Qiagen, Hilden, Germany) per the manufacturer’s instructions. A qPCR to detect SPV was run as previously described [12] in a 20 µL reaction volume containing 6.7 µL of DNAse/RNAse-free water, 400 nm of each forward and reverse primer (Table S1), a 250 nm probe, 1X of 2X iQ Supermix (Bio-Rad, Waltham, CA, USA), and 2 µL template DNA. The qPCR reaction was performed using a CFX96 Touch Real-Time PCR Detection System (Bio-Rad) with cycling conditions as follows: initial denaturation at 95 °C for 10 min, then 45 cycles of 95 °C for 15 s, annealing at 60 °C for 60 s, extension at 72 °C for 30 s, and a final extension at 72 °C for 5 min. A sigmoidal curve peak with a *Cq* value < 35 indicated a positive amplification result, a *Cq* > 35 was considered inconclusive, and no amplification peak was considered negative [27].

### 2.4. Amplification and Sequencing of Selected Capripoxvirus Genus-Specific Marker Genes

The SPV-positive samples were further characterized through the amplification and sequencing of four *Capripoxvirus* genes: the RNA polymerase 30 kDa subunit (RPO30), the G-protein-coupled receptor (GPCR), extracellular enveloped virus (EEV) glycoprotein, and the *Capripoxvirus* homolog of the variola virus B22R gene. These genes were amplified as previously described [28,29,30,31] in a 20 μL PCR reaction volume containing 500 nM of each of the forward and the reverse primers, 200 µM of dNTPs, 1x PCR buffer (Qiagen), 1.25 U of Taq DNA polymerase (Qiagen), and 2 μL template DNA. Positive amplicons were sent for sequencing at LGC Genomics (Berlin, Germany).

### 2.5. Sequence and Phylogenetic Analysis

The sequences were cleaned and assembled using the Vector NTI software (Invitrogen, Waltham, CA, USA) version 11.5. Multiple sequence alignments of good-quality sequences for each of the targeted genes, together with representative *Capripoxvirus* sequences retrieved from GenBank, were performed on MEGA X (version 10.1 8) using the muscle algorithm and the codon option. Maximum-likelihood trees were constructed on MEGA X using the complete RPO30 and GPCR gene sequences, with evolutionary distances computed using the Hasegawa, Kishino, and Yano (HKY) and General Time-Reversible (GTR) models, respectively, with gamma-rate distributions and 1000 bootstrap replicates. The model for each dataset was selected after computation of the Best-Fit Substitution Model on using based on the BIC (Bayesian Information Criterion) score. The phylogenetic trees were visualized using the Interactive Tree of Life (ITOL) tool, while the multiple sequence alignments of the RPO30, partial EEV glycoprotein, and B22R genes were visualized using BioEdit (v7.2.6).

## 3. Results

**Figure 2:**
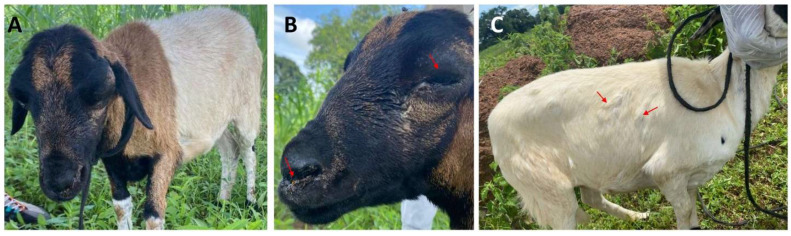
Clinical signs observed in suspected sheep from a farm in Vunania. (**A**) lethargic sheep; (**B**) sheep with swollen eyes and oculonasal discharges; (**C**) generalized pox-like lesions on the sheep (red arrows).

A provisional diagnosis of SP was established through physical examination (Figure 2) and clinical history. The ages of the affected sheep ranged from 3 months to 4 years, encompassing breeds such as Nungua Blackhead, West African Long Legged, and domestic crossbreeds. The clinical manifestations observed included dyspnea, hyperthermia, oculonasal discharges, lethargy, cutaneous ulcerations, and pox-like lesions at various developmental stages (Figure 2). These symptoms were indicative of SP [4,15,16] and were identified in 76.1% (51/67) of the sheep across the four farms from which samples were collected, with a mortality rate of 37.3%. Sheep exhibiting severe symptoms received symptomatic treatment, including antibiotics for suspected secondary infections, multivitamins, and fluids. A majority (62.7%) of the remaining sheep recovered without intervention.

**Figure 3:**
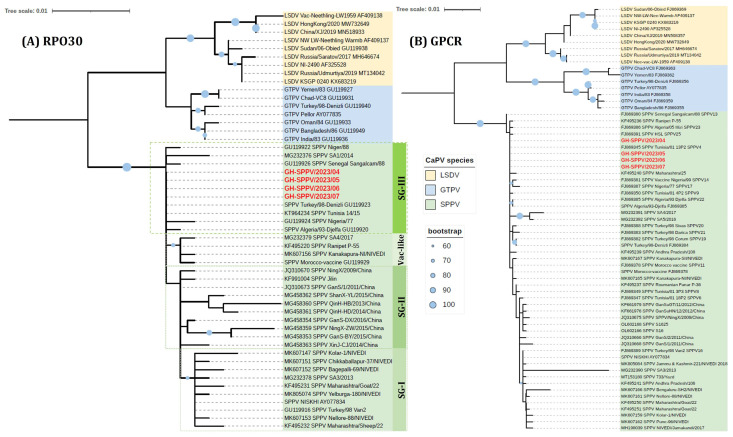
Maximum-likelihood phylogenetic trees of *Capripoxviruses* (CaPV) with SPVs from Ghana based on (**A**) complete RPO30 gene sequences, applying the HKY model and gamma-rate distributions, and (**B**) complete GPCR gene sequences, applying the GTR model and gamma-rate distributions. The trees are visualized in iTOL with SPVs from Ghana (in red). Note: Previous abbreviations (i.e., SPPV and GTPV) have been used in the sequence names.

**Figure 4:**
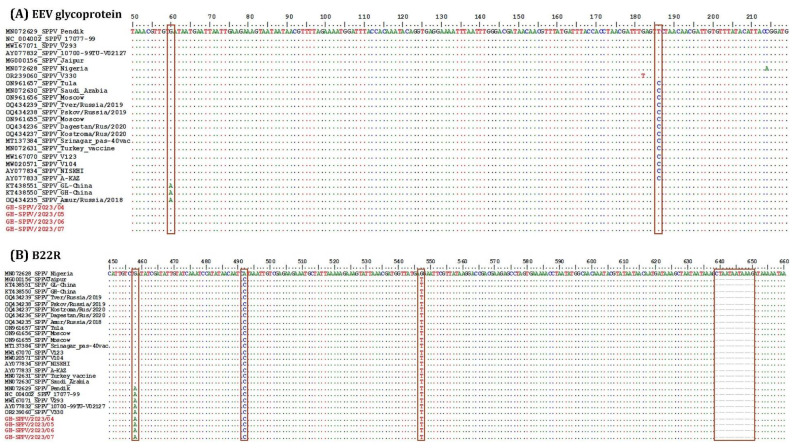
Multiple sequence alignment of (**A**) EEV glycoprotein gene and (**B**) B22R gene. Ghana SPV sequences (in red) were aligned with representative SPV sequences retrieved from GenBank for each gene. The main SNPs and deletions in the alignment are shown in the blocks. The dots indicate identical nucleotides in the alignment. Note: The previous abbreviation (i.e., SPPV) has been used in the sequence names.

Of the 12 samples collected, 11 tested positive for SPV. Subsequently, four samples exhibiting the lowest Cq values [GH-SPPV/2023/04 (Cq:29.27), GH-SPPV/2023/05 (Cq:23.58), GH-SPPV/2023/06 (Cq:35.74), and GH-SPPV/2023/07 (Cq:33.27)] were selected for sequencing. To characterize the SPV circulating in sheep in Ghana, the RPO30, GPCR, EEV glycoprotein, and B22R genes were sequenced and compared to other global isolates using multiple sequence alignment and phylogenetic analysis (Figure 3 and Figure 4). Following quality control and sequence editing, the targeted genes were found to be identical across all four samples. Consequently, GH-SPPV/2023/04 was chosen as a representative sample and submitted to the GenBank database under accession numbers PV577422, PV577423, PV577424, and PV577425 for the RPO30, GPCR, EEV glycoprotein, and B22R genes, respectively. The phylogenetic tree of RPO30 revealed three subgroups of SPVs, SGI, II, and III, predominantly comprising Indian/Turkish, Chinese, and African isolates, respectively. The Ghana SPV sequences clustered in SGIII with other African SPVs, including those from Senegal, Algeria, Tunisia, Morocco, and Nigeria (Figure 2A). Clustering of SPVs based on the RPO30 gene phylogenetic tree was also observed in both the nucleotide and amino acid multiple sequence alignments of the RPO30 sequences (Supplementary File S1: Figure S1). The GPCR phylogenetic tree clustering of SPVs was less distinct than that of the RPO30 analysis; however, the Ghanaian SPV sequences grouped with other African SPVs and were 100% identical to SPV from Tunisia (GenBank# FJ869345) (Figure 3B). Additionally, EEV glycoprotein and B22R fragment sequence alignment indicated that Ghana SPVs were similar to SPV isolates from Turkey (MN072629, NC_004002, AY077832), Egypt (MW167071), and the UAE (OR239060).

## 4. Discussion

This study represents the first identification and characterization of SPV in sheep in the northern region of Ghana, a finding that has not been previously documented. This presents a considerable challenge to veterinary authorities in Ghana, as the disease is not included in the nation’s list of notifiable diseases on account of being considered absent. However, our findings present a unique opportunity for sheep pox to be considered for surveillance and notification.

Ghana participates in livestock trade along its northern border with Burkina Faso and other West African nations. Although it remains uncertain whether SPV was introduced through trade, the dynamic movement of animals may have facilitated the virus’s dissemination across Africa. This pattern of spread, enabled by established transhumance activities and animal trade between West Africa and Sahelian Africa [32,33], has been documented for the transmission of FMD type O from West Africa to the Maghreb region in 1999 [34] and for the spread of PPR lineage IV in West Africa [35]. The observed genetic relatedness of GPV and LSDV from Ghana to isolates from other African countries [36,37] suggests that these viruses may have been introduced into outbreak locations via transhumance activities and live animal trade. This hypothesis is supported by the presence of a robust live ruminant market and trade in the region, which imports ruminants from neighboring West African countries and distributes them throughout Ghana.

The endemicity of SPV can be assessed using mortality and morbidity rates, which are inversely related to the endemicity of the disease [38,39] and are correlated with the presence of anti-SPV antibodies acquired through infection [21]. In endemic regions, the majority of sheep are expected to recover within approximately three weeks without systemic treatment [6,7,40]. Furthermore, only a limited number of sporadic suspected SP outbreaks have been diagnosed in Ghana [41] or anecdotally identified based on pathognomonic signs. Consequently, it is plausible that SP is not endemic in Ghana and that the current outbreak was sporadic in a naïve sheep population, particularly given its high infectivity and relatively elevated mortality rates. The management of the outbreak did not adhere to practices recommended for “naïve” regions. Specifically, there has been no implementation of ring vaccination, culling of infected animals, or establishment of 3 km protection or 10 km surveillance zones, as recommended for outbreak situations [4,7,8]. SPV-based vaccines have been demonstrated to be effective [7,13,21,22,23,24,25], long-lasting, lifelong [4,11], and safe [21,22,23]. However, because of SPV’s exclusion from Ghana’s list of notifiable diseases and its lack of prioritization, vaccination has not been implemented.

This study provides molecular evidence for the presence of SPV in northern Ghana. It is recommended that the Veterinary Service Directorate of Ghana consider revising the list of notifiable diseases to include SP, enabling the implementation of specific surveillance, control, and management programs for the disease.

## Data Availability

The sequences generated in this study have been submitted to GenBank under accession numbers PV577422 to PV577425.

## Supplementary Materials

The following supporting information can be downloaded at https://www.mdpi.com/article/10.3390/v17070875/s1: Supplementary Figure S1: The clustering of SPVs based on the RPO30 gene phylogenetic tree was also observed in both nucleotide and amino acid multiple sequence alignments of the RPO30 sequences; Table S1: Genes used for the PCR identification of Capripoxvirus sheeppox.
