# Supplementary material for: First Molecular Characterization of Sheep Pox Viruses in Northern Ghana, 2023
Source: Viruses. 2025 Jun 21;17(7):875. doi: 10.3390/v17070875 (PMC12300133; doi:10.3390/v17070875)
Supplement: Supplementary file 1 [file viruses-17-00875-s001.zip › viruses-3665299-supplementary.pdf]

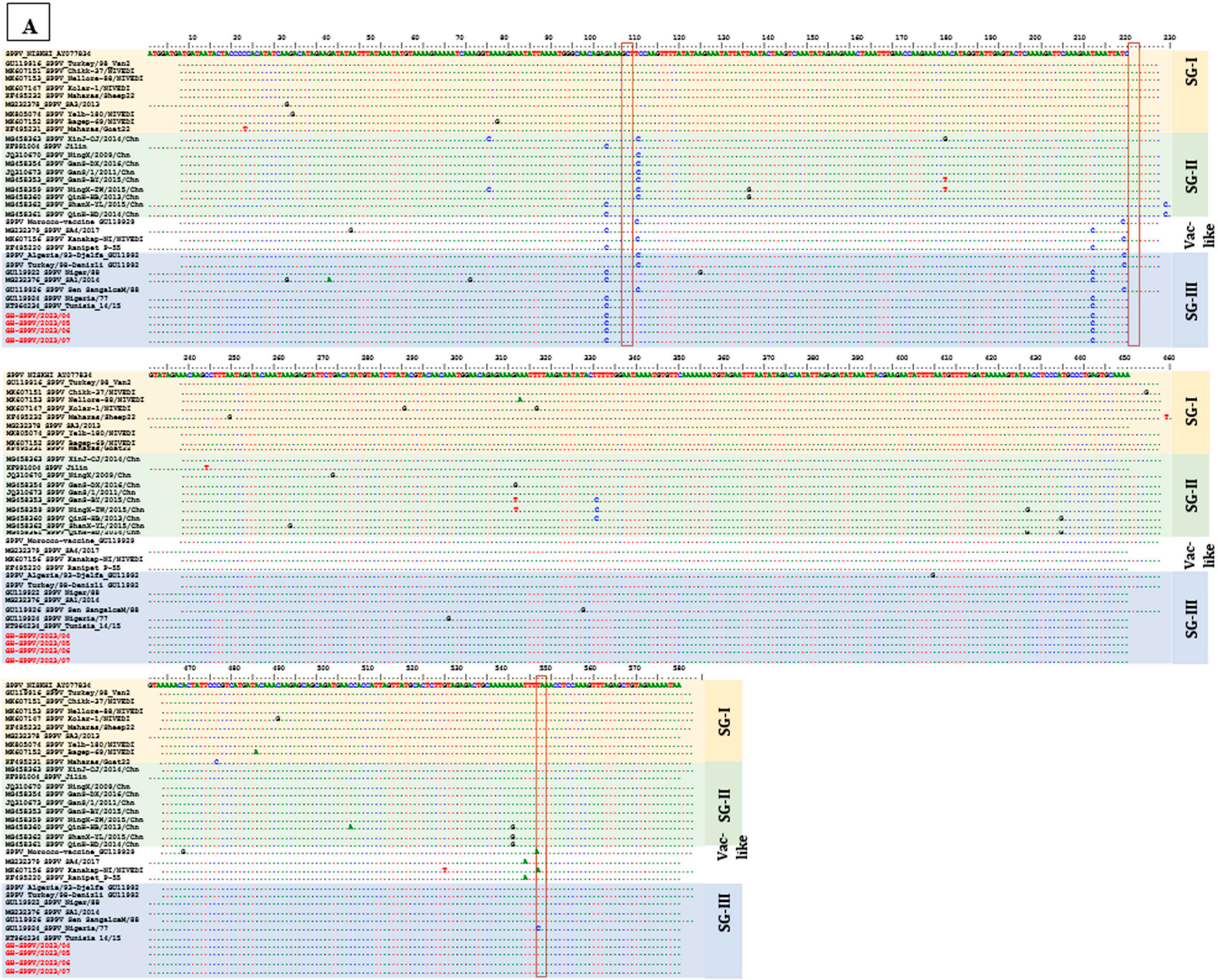
A
SG-I
SG-II
Vac-like
SG-III

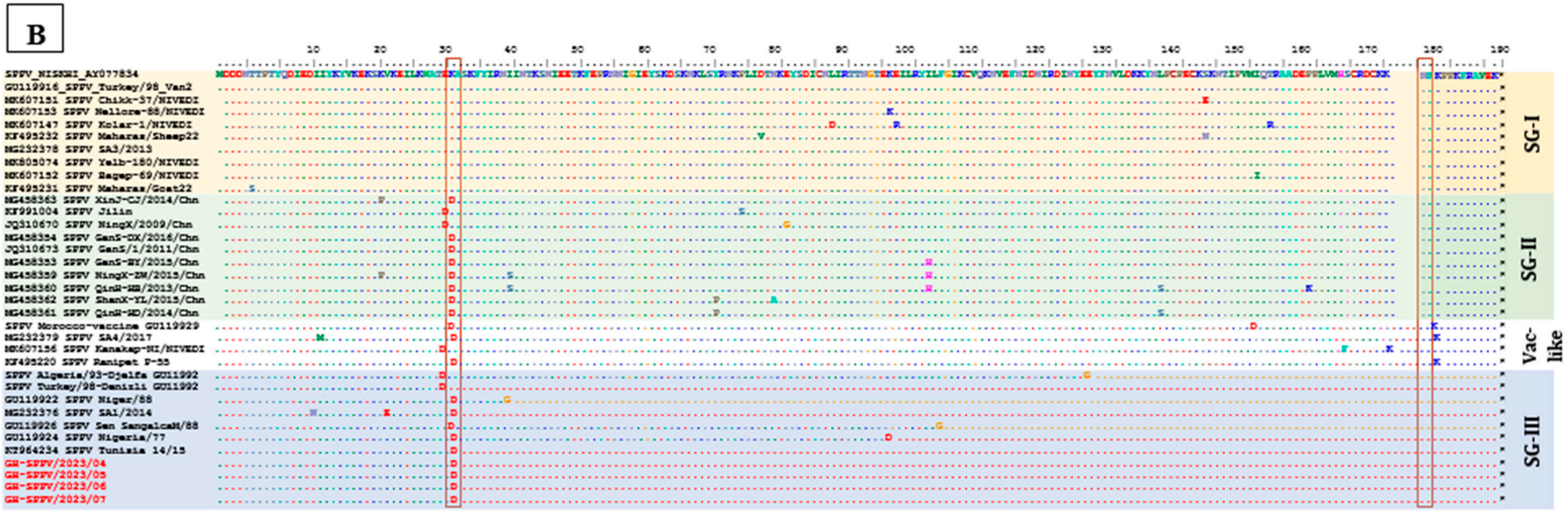

Supplementary Figure S1: The clustering of SPVs based on the RPO30 gene phylogenetic tree was also observed in both nucleotide and amino acid multiple sequence alignments of the RPO30 sequences

Supplementary Table S1: Genes used for the PCR identification of capripoxvirus sheeppox

**Item description**	**Company/Cat No**	**Storage**
CaPV074F1: (Forward primer) -20 µM AAAACGGTATATGGAATAGAGTTGGAA	Applied Biosystems, Eurogenetec	Store the diluted primers and probes at -20°C
CaPV047R1: (Reverse primer) -20 µM AAATGAAACCAATGGATGGGATA		
CaPV074P1: (Probe) – 10 µM FAM-TGGCTCATAGATTTCCT-MGB/NFQ		
iQ Supermix	Thermo fischer scientific	- 20°C
